# Wound Dressing Based on Cassava Silk-Chitosan

**DOI:** 10.3390/ma17122986

**Published:** 2024-06-18

**Authors:** Yumei Chen, Haitao Lin, Xinxia Yue, Enping Lai, Jiwei Huang, Ziyu Zhao

**Affiliations:** 1 School of Guangxi Key Laboratory of Sugar Resources of Green Processing, School of Biological and Chemical Engineering, Guangxi University of Science and Technology, Liuzhou 545006, China; 644712639@163.com (Y.C.); xinxiayue@126.com (X.Y.); nemodhu@163.com (E.L.); huangjiwei@gxust.edu.cn (J.H.); 2 School of Engineering Research Center for Knitting Technology, Ministry of Education, Jiangnan University, Wuxi 214122, China

**Keywords:** cassava silk, chitosan, sponges, antimicrobial, drug delivery

## Abstract

The application prospects of composite sponges with antibacterial and drug-carrying functions in the field of medical tissue engineering are extensive. A solution of cassava silk fibroin (CSF) was prepared with Ca(NO_3_)_2_ as a solvent, which was then combined with chitosan (CS) to create a sponge-porous material by freeze-drying. The CSF-CS composite sponge with a mesh structure was successfully fabricated through hydrogen bonding. Scanning electron microscopy (SEM), Fourier transform infrared absorption (FTIR) and X-ray diffraction (XRD) were employed to investigate the appearance and structure of the cassava silk’s fibroin materials, specifically examining the impact of different mass percentages of CS on the sponge’s structure. The swelling rate and mechanical properties of the CSF-CS sponge were analyzed, along with its antibacterial properties. Furthermore, by incorporating ibuprofen as a model drug into these loaded sponges, their potential efficacy as efficient drug delivery systems was demonstrated. The results indicate that the CSF-CS sponge possesses a three-dimensional porous structure with over 70% porosity and an expansion rate exceeding 400% while also exhibiting good resistance against pressure. Moreover, it exhibits excellent drug-carrying ability and exerts significant bacteriostatic effects on Escherichia coli. Overall, these findings support considering the CSF-CS composite sponge as a viable candidate for use in drug delivery systems or wound dressings.

## 1. Introduction

The inflammatory period of wound healing is highly susceptible to bacterial infection, which can lead to life-threatening complications. Therefore, appropriate dressing is essential for effective wound care. Nowadays, functional dressing has become a research hotspot in clinical medicine [1]. In recent years, sponges have been extensively investigated for their applications in tissue engineering and biomedical fields such as wound dressings, drug release control, and in vivo implantation [2]. Sponges are composed of natural polymers that are highly hydrophilic and biocompatible, containing a binary or ternary macromolecular network structure [3,4,5,6].

Function wound sponges need similarity to the extracellular matrix and good acceptance by biological systems. Naturally occurring polymers have attracted increased attention for their potential applications as the wound dressing materials due to their unique biological activities [7]. For example, S. Alven. et al. prepared a sponge based on gelatin, polyethylene glycol, and Ag, which has good antibacterial properties and allows for long, sustained release of loaded drugs [8]. Y. Liang. et al. prepared a hydrogel based on chitosan-grafted dihydrocaffeic acid and oxidized pullulan, which has fast gelation time, excellent antimicrobial properties, drug-carrying properties, and the potential for local drug delivery. However, its preparation process is complicated and time-consuming [9]. J. Leppininemi. et al. described a hydrogel based on nanocellulose and alginate for 3D printing, using NHS and EDC crosslinking. This hydrogel exhibits great compressive stress and cellular compatibility. However, the addition of crosslinking agents during the preparation process increases the cost and prevents large-scale production [10].

The primary structure of cassava silk is mainly composed of approximately 100 repeats of alternating polyalanine Ala- and Gly-rich structural domains [11,12]. The glycine-rich gene sequences are essentially in random coil states, providing elasticity to the silk fibers, while the crystalline regions form a β-sheet conformation [13,14]. The regular polypeptide sequences of (-Ala-)n in cassava silk could further enhance the elastic modulus and breaking strength of cassava silk-based biomaterials [15]. Cassava silk fibroin (CSF) is a natural protein polymer extracted from cassava silk, exhibiting excellent biocompatibility, mechanical properties and a slow degradation rate. Therefore, CSF can be easily processed into films [12,16], sponges [17], microparticles [18], nanofibers [19] and scaffolds [20]. In addition, previous studies have shown that CSF also contains the Arge-Gly-Asp acid (RGD) tripeptides, which promote rapid wound healing [21,22,23,24]. The cassava silkworm is also the only wild silkworm that can be completely domesticated. Based on its advantages, CSF serves as an effective wound dressing material.

Chitosan (CS) is a cationic polysaccharide material characterized by the presence of amino groups, offering hemostatic antimicrobial properties [25]. It has good antimicrobial, biocompatible and biodegradable properties [26,27], and its good effect in accelerating wound healing and hemostasis makes it an ideal material for the new generation of medical dressings [28,29]. In addition, studies have demonstrated that CS can induce fibroblast proliferation by promoting neutrophil migration, expediting wound healing and preventing wound infections [9,30,31,32]. However, the mechanical properties of CS sponges are poor and their applicability is limited. To enhance their performance, they are frequently blended with additional polymers or supplemented with other chemical moieties. It has an excellent drug release rate, but its preparation is complex and time-consuming. To address these shortcomings, sustainable and cost-effective technologies need to be developed.

In this study, we aimed to design a multi-functional sponge with antimicrobial and degradability, serving as a localized drug carrier. The preparation time of the sponge is short, and no crosslinker is required. The sponge was prepared by adding different mass fractions of CS to CSF. The internal structure, surface porosity, thermal properties, swelling rate, mechanical properties and enzymatic degradation properties of the sponges were evaluated. The anti-inflammatory drug ibuprofen (IBU) was used as a model drug for drug release experiments in a simulated in vitro environment. The bacteriostatic effects on *E. coli* and *S. aureus* were tested in vitro. The intrinsic properties and biological findings of the sponges open up their potential application for biomedical applications and drug-delivery carrier systems.

## 2. Materials and Methods

### 2.1. Materials

Cassava silk (Guangxi Silk Center), chitosan (Mn = 100,000–300,000 Da, Macklin, Shanghai, China), calcium nitrate tetrahydrate (4H_2_O∙Ca (NO_3_)_2_, >98%), Streptavidin E (Macklin, Shanghai, China), Staphylococcus aureus (*S. aureus*), Escherichia coli (*E. coli*), NaHCO_3_ (>98%), KCl (>98%), NaCl (>98%), Na_2_HPO_3_ (>98%), K_2_HPO_3_ (>98%), (all from Macklin, Shanghai, China).

### 2.2. Methods

#### 2.2.1. CS Solution

Three g of CS was dissolved in 100 mL of a 3 wt% acetic acid solution. The solution was stirred until it was completely dissolved and then allowed to defoam. Finally, solution was stored in a refrigerator at 4 °C.

#### 2.2.2. Cassava Silk Solution

The cassava silk fibroin was boiled in a 0.5 wt% Na_2_CO_3_ solution three times, thoroughly rinsed with water to remove the silk glue and then left to air dry at room temperature. The fibroin was dissolved by a 4H_2_O∙Ca(NO_3_)_2_ solution at a concentration of 10% mass, heated at 110 °C for 3 h [15].

The fibroin solution was dialyzed against deionized water (pH = 8) for at least 2 days using dialysis cassettes (Pierce Snake Skin MWCO 8000-12000, JielePu, Chicago, IL, USA). The dialysis cassettes containing the CSF solution were placed into polyethylene glycol (PEG) for concentration over a period of 12 h and stored at 4 °C for preservation.

#### 2.2.3. CSF-CS Sponge Preparation

**Figure 1:**
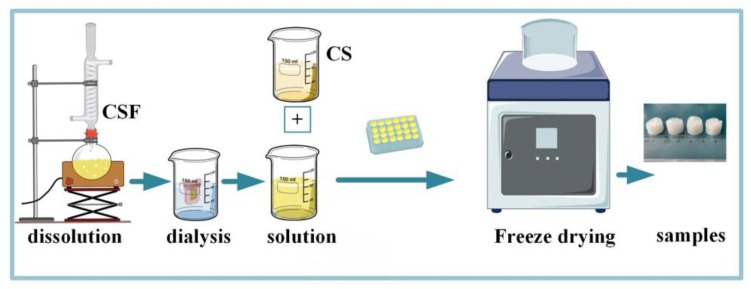
The sketch of the experimental process for the CSF-CS sponge preparation.

The cassava silk solution with a mass fraction of 3% and the CS solution were mixed well in different mass percentages. The solutions were mixed well by magnetic stirring and then transferred to 24-well plates at 2 mL per well. After pre-freezing at −20 °C for 8 h, the plates were placed in a −60 °C refrigerator to continue freezing for 12 h, and then vacuum freeze-dried for 48 h to obtain regular sponges. After freeze-drying, the sponges were collected and sealed and placed in the refrigerator at 4 °C for storage. A sketch of the experimental process is shown in Figure 1. 

### 2.3. Physical Property Characterization

#### 2.3.1. Fourier Transform Infrared Spectroscopy (FTIR)

FTIR spectra of the different mass percentages of CSF-CS sponges were recorded from 650 cm^−1^ to 4000 cm^−1^ using a Nicolet S10 FTIR spectrometer (Thermo Scientific Instruments, Waltham, MA, USA). 

#### 2.3.2. X-ray Diffraction (XRD)

The samples were ground into powder, and the powdered samples were characterized using an X’Pert-Pro fully automated X-ray diffractometer (Bruker, Billerica, CA, USA). The diffractograms of the samples were recorded with Cu Kα radiation (λ = 1.542) in a 2θ angle ranging from 5° to 60° at 40 kV and 40 mA.

#### 2.3.3. Scanning Electron Microscope (SEM)

The sponges were routinely coated with gold film, and the morphology of the stents was analyzed by a Feiner scanning electron microscope (Sigma300, Oberkochen, Germany). The samples were subjected to aperture analysis using Image J software (v. 1.54f). The average pore size was obtained by measuring the sample and the maximum and minimum diameters of at least 10 randomly selected pores in the central portion. 

#### 2.3.4. Thermal Properties

The thermal properties of the CSF-CS composite sponges were analyzed by thermogravimetric analysis. Thermogravimetric analysis was carried out by Thermal Analyzers (Q5000 TA, New Castle, DE, USA). The samples were heated from 25 to 600 °C under a nitrogen atmosphere at 10 °C × min^−1^. 

#### 2.3.5. Porosity

The porosity of the composite sponge was determined using the pycnometer method. After cutting the samples to a uniform size, absolute ethanol was added to a specific gravity flask. The weight of the Pyrex containing absolute ethanol was measured (W_1_). Subsequently, the dried sponge (W_0_) was immersed in absolute ethanol and subjected to vacuuming for 30 min to ensure complete impregnation with ethanol. Ethanol was then added until the scale on the Pyrex reached its original level, and the weight was recorded (W_2_). Finally, the samples were removed and weighed again to determine the remaining amount of ethanol (W_3_) [33].

Porosity (%) = [(W_2_ − W_3_ − W_0_)/(W_1_ − W_3_)] × 100

#### 2.3.6. Swelling Rate

The water-absorbing swelling rate of each group of stents was determined using the conventional weight method. First, the freeze-dried sponges were weighed (Wd) and then immersed in phosphate buffer solution (PBS, pH = 7.4) for 10 h. After removing excess water from the surface of the sponges using absorbent paper, the wet weight (W_s_) of the swollen sponges was recorded. The determination of the water absorption rate was based on the disparity between the swollen state (after reaching equilibrium and eventual degradation or partial dissolution) and the initial dry weight.

Swelling (%) = [(W_s_ − W_d_)/W_d_] × 100

#### 2.3.7. Mechanical Properties

The compression mechanical properties were tested using a small tensile strength machine. The speed of the horizontal head was set to 2 mm/min, and the compression was set to 80%. Stress–strain curves were then plotted to assess the mechanical properties of the porous composite stents, and the slope of the initial linear phase of the stress–strain curve was the modulus of elasticity.

Stress: σ = F/A; Strain: ε = ΔL/L_0_ × 100%

#### 2.3.8. Enzymatic Degradation Properties

To evaluate the enzyme degradation behavior of the sponge, samples were used to evaluate the degradation behavior of the sponge according to the percentage of residue after incubation in the phosphate buffer containing the enzyme. The quality of the sponge was recorded after vacuum drying (Wf), and then the sponge was soaked in PBS (pH = 7.4) containing 1 u/mL Streptomycin E (Macklin, Shanghai, China) at 37 °C for a certain time. The samples were taken out at the specified time interval, vacuum dried for 24 h, weighed and recorded (Wt). The experiment was carried out three times, and their average values and their respective standard deviations were taken.

Mass remaining (%) = [(Wt − Wf)/Wf] × 100

### 2.4. Bacteriostatic and Drug Release

#### 2.4.1. Drug Release

The drug release rate of CSF-CS composite sponges was investigated using IBU as a model drug for drug loading and in vitro release studies. The samples were immersed in anhydrous ethanol containing 10 mg/mL of an IBU solution for 12 h. Afterward, the immersed samples were frozen at −60 °C for 4 h and freeze-dried for 3 days. 

The weight of the IBU-loaded sponge was measured, and it was placed in a centrifuge tube with 15 mL of PBS (pH = 7.4). The tube was then placed in a constant temperature bath and shaken at 37 °C and 60 rpm. At predetermined time intervals, 4 mL of the release solution was removed and returned to an equal volume of fresh PBS. The samples were analyzed by UV–visible spectroscopy at 263 nm in a UV–2700i (Shimadzu, Kyoto, Japan). A standard curve was configured and measured (concentration range 0.0 to 400 μg/mL) to calculate the concentration of IBU, correlating the amount of IBU released to the intensity of absorbance.

#### 2.4.2. Antibacterial Tests

Sponge extracts of Gram-negative species of *E. coli* and Gram-positive species of *S. aureus* were used to evaluate antimicrobial activity. CSF, CSF-CS20%, CSF-CS40% and CSF-CS60% were set as the experimental group, and the blank surface was taken as the blank group.

100 μL of *E. coli* or *S. aureus* inoculum were added to 100 mL of nutrient broth water to make a bacterial solution. 0.25 g of sterilized specimen was added to this bacterial solution. A bacterial solution without a sample was used as a blank group. The inoculated samples were incubated in a shaker bed for 12 h at 37 °C and 200 rpm. Then, the bacterial solution after 12 h of shock culture was taken from the conical bottle of the experimental group and the blank group, respectively, for 10^−6^ times gradient dilution. A total of 50 μL of the extract mixture was plated on agar plates and incubated for a further 18 h at 37 °C. After incubation, the plates were observed on the light, and pictures were taken. The antibacterial tests for the respective samples were repeated up to three times. Hence, we have reported the results from only one of the reproducing studies.

Antibacterial activity = [Number of bacteria on blank sample − Number of bacteria on the sample]/[Number of bacteria on blank sample]

## 3. Results and Analysis

### 3.1. Physical Properties

#### 3.1.1. Chemical Characterization of the Composite Sponges

**Figure 2:**
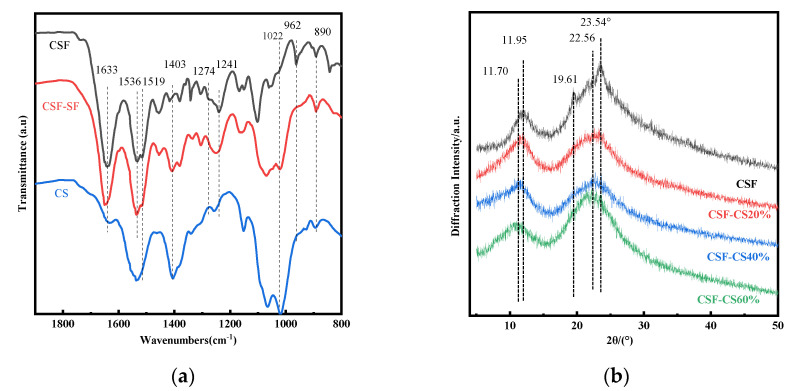
(**a**) FTIR of the CSF, CSF-CS and CS sponges. (**b**) XRD of the different mass percentages of CSF-CS composite sponges.

The FTIR of CSF, CS and CSF-CS composite sponges in the range of 1900–800 cm^−1^ (Figure 2a) were conducted to investigate the structural changes. The relationship between the peak spectral bands and the β-sheet structure was summarized [34,35,36]. The FTIR showed that the CSF sponges had peaks at 1633 cm^−1^, 1519 cm^−1^ and 1241 cm^−1^, all of which are typical peaks characteristic of β-sheet and randomly curled conformations (Figure 2a). When comparing CSF with the spectra of CSF-CS and CS, a shift in the characteristic absorption peaks was observed: 1630–1635 cm^−1^ (amide I), 1516−1521 cm^−1^ (amide II), 1240–1235 cm^−1^ (amide III) and 965 cm^−1^ (amide IV) correlate with the intermolecular β-sheet bands [26]. CS not only induces the formation of β-sheet structures but also promotes the formation of intermolecular–intramolecular networks into the CSF matrix skeleton. This indicates that the CSF structure changes from a random coil to a β-sheet structure [37]. The crystalline structure of the CSF-CS porous sponge was analyzed by XRD. The XRD curve is shown in Figure 2b. The XRD of wild silk fibroin was determined in earlier studies as follows: 11.95°, 24.02°, for Silk I (contains β-sheet and α-helix), and 16.71°, 20.34°, 24.49°, 30.90°, 34.59°, 40.97° and 44.12° for Silk II (mainly β-sheet) [38,39,40] Based on these studies, the pure CSF sponges have a strong peak at 11.95° and 23.54°, which belong to the Silk I structure (Figure 2b). There are two minor peaks of about 19.61° and 22.56°, which belong to the Silk II structure. Thus, the pure CSF sponges contain a small amount of α-helical structure and a large amount of β-sheet structure. Regarding the mass percentage of CSF-CS, the peaks of 11.70° and 22.56° were shifted to a lower angle, the peaks of 19.61° was covered, and there were two major peaks of about 11.70° and 22.56°, which belong to the Silk II structure. Therefore, it can be concluded that CSF-CS sponges induce a fundamental rearrangement of molecular conformation, transforming α-helix and random coil structures into β-sheet. 

#### 3.1.2. Scanning Electron Microscopy Observation (SEM)

**Figure 3:**
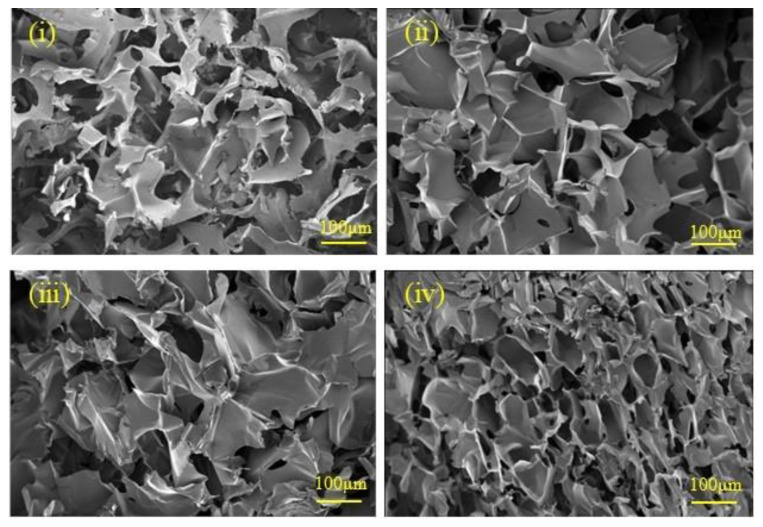
SEM of the different mass percentages of CSF-CS composite sponges: (**i**) CSF, (**ii**) CSF-CS20%, (**iii**) CSF-CS40% and (**iv**) CSF-CS60%.

The SEM images of each group of sponges are presented in Figure 3. The CSF-CS composite sponges exhibited a porous structure, with the average pore size of the pure CSF sponges being the largest at 126.2 μm, while that of the CSF-CS 60% sponges was the smallest at 60.2 μm. When the mass percentages of CS and CSF within the sponges were varied, the sponges were able to maintain a highly porous network structure and good inter-pore connectivity. With the increase in the mass percentage of CS in the sponges, the pore diameters of the sponges decreased. When CS was 40% or above, the pore diameters of the porous sponges could be clearly seen to be decreased. This is attributed to the fact that CS promotes the irregularly curled structure of CSF to the β-sheet structure transformation, which makes the pore size of the sponges decrease.

#### 3.1.3. Thermogravimetric Analysis

**Figure 4:**
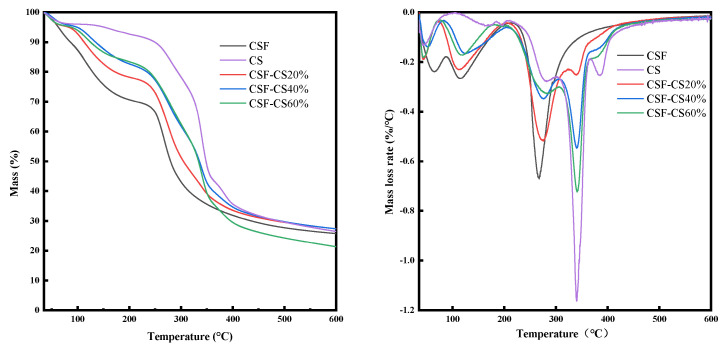
TG and DTG in 35–600 °C for the different mass percentages of CSF-CS composite sponges.

As shown in Figure 4, the thermogravimetric curves of the mass percentages of CSF-CS sponges may be divided into three stages. In the first stage of 35 to 250 °C, mass loss is attributed to the removal of adsorbed water from the materials. In the second stage (about 250–360 °C), there is an accelerated mass loss rate as compared to the first stage, indicating the decomposition of the sponge material. In the third stage (over 338 °C), the decomposition rate further accelerates. 

As shown by the thermogravimetric curves, it is obvious that the mass percentage of loss increases with an increase in the mass percentage of CS in the first stage. In the presence of more mass percentage of CS, more water was adsorbed by the sponge, and so a large amount was lost during the heating process. In the second stage, the mass loss rates of the samples were greater than those detected for pure CSF. During the decomposition process, cassava silk formed β-sheet structure under the induction of chitosan, but the change of the β-sheet structure content had no significant effect on the main stage of thermal decomposition. Finally, in the third stage (over 338 °C), the reduced thermal stability of SF as compared to CS induced greater mass loss in the sponge samples containing higher proportions of CSF. Therefore, the curve indicates that the thermodynamic properties of the CSF-CS sponges were enhanced by the transition of the β-sheet structure.

#### 3.1.4. Porosity and Swelling Rate

**Figure 5:**
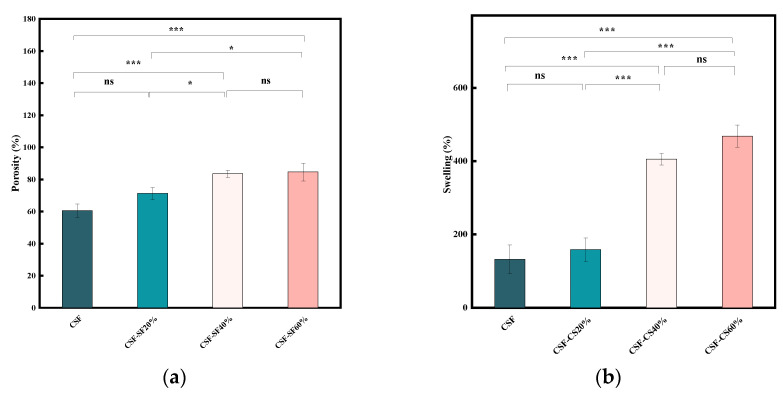
(**a**) Porosity of different mass percentages of CSF-CS composite sponges. (**b**) Swelling of different mass percentages of CSF-CS composite sponges. Comparisons of data from independent samples were performed using the *t*-test, and repeated measurements were tested using the p-test, and *p* < 0.05 was considered statistically significant. Confidence level is labeled as: * indicates *p* < 0.05, and *** indicates *p* < 0.001, ns indicates no significance.

The porosity of the CSF-CS composite sponges is shown in Figure 5a and Table 1. The pure CSF sponges exhibited the highest porosity at about 60.5 ± 4.2%. The porosity of the CSF-CS20% sponges was about 71.2 ± 3.7%, which may be due to the fact that CS induces CSF’s crosslinking matrix, and the sponges were mainly CSF-based. The sponges of CSF-CS40% and CSF-CS60% had similar porosities, 83.0 ± 2.4%, and 86.7 ± 5.6%. As indicated in Table 1, all of these sponges showed a porosity exceeding 70%. When the CS mass fraction was 40%, the sponges were still dominated by the CSF matrix internally, resulting in higher porosity. However, when the CS mass fraction was 60%, the internal structure was mainly dominated by CS, and thus the internal porosity did not decrease despite the reduced CSF content. At a CS content of 40%, the primary structure involved CS inducing the formation of a β-sheet structure, leading to a decrease in average pore size and an increase in porosity. When the CS content increased to 60%, the excess CS participated in the formation of the pore wall, resulting in a small increase in porosity.

The swelling of the sponges is crucial for tissue engineering, as it affects cell migration, proliferation and differentiation, as well as nutrient transport and mechanical properties [41]. Figure 5b illustrates the swelling rate of the CSF-CS sponges, which was statistically significant across all four sample groups. The swelling of pure CSF sponges was poor, only 132%. While the swelling of the CSF-CS composite sponges increased proportionally with an increasing mass percentage of CS, there was minimal variation in the swelling rate, which was between 40% and 60% CS. This suggests that enhanced swelling resulting from an increase in the mass percentage of CS is facilitated by the crosslinking matrix present within both CSF’s mesh structure and CS. However, it should be noted that excessive swelling properties are limited by CSF’s mesh structure and the crosslinking matrix of CS. Ideal wound dressings should possess various properties including a hydrophilic three-dimensional structure, high water content, appropriate pore size and porosity, good biocompatibility and excellent mechanical properties. These combined characteristics create an optimal microenvironment for cell growth and drug delivery while ensuring high strength in wound dressings.

Porous sponges with a porosity greater than 70% are considered to be good spaces for cell survival and nutrient transportation channels for maintaining cell growth [42]. Highly porous structures can also provide sufficient nutrient and gas exchange and enough space for cell proliferation and attachment [41,43]. The nutrient transport capacity of the sponges is closely related to the porosity, pore size and pore connectivity of the sponges. Both the porosity and swelling rate have important effects on drug loading. Therefore, porosity and swelling are important characteristics of tissue engineering sponges.

#### 3.1.5. Mechanical Strength and Enzymatic Degradation Rate

**Figure 6:**
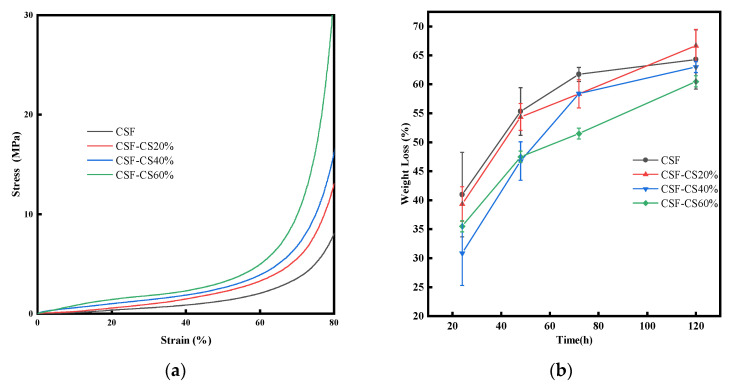
(**a**) Stress–strain curves for different mass percentages of CSF-CS sponges at 80% compression. (**b**) Enzymatic degradation rates for different mass percentages of CSF-CS composite sponges.

Mechanical strength is a critical factor in evaluating the material properties of medical dressings. The compressive stress–strain curve is shown in Figure 6a. The mechanical properties of the sponges were assessed by comparing their compressive stress–strain curves. The compressive strength of the pure CSF sponges was the lowest, and that of the CSF-CS60% sponges was the highest. The strength of the CSF-CS sponges increased with the increase in the mass percentage of CS, and the high mass fraction of CS helped to improve strength. The mechanical properties of the CSF-CS crosslinked matrix were enhanced with an increase in the CS mass fraction, which was attributed to the β-sheet structure induced by CS. However, at 60% of CS, there was a decrease in the β-sheet structure formation within the CSF-CS matrix. The SEM image revealed that the internal structure of the CSF-CS60% sponge exhibited fewer pores than the CSF skeleton and more pores formed by CS. Consequently, while mechanical characteristics and increasing porosity were enhanced, there were no significant differences observed in the swelling rate between CSF-CS60% andCSF-CS40%. Sponges with specific mechanical properties can provide support while possessing sufficient capacity to withstand repeated dynamic shocks without collapsing or deforming. The compressive strength of pure CSF is low. With the increase in CS content, the compressive performance of the sponge is gradually increased, and it has better compressive strength and durability.

In this investigation, the enzymatic degradation of CSF-CS sponges was investigated in vitro. As shown in Figure 6b, the sponges containing CSF exhibited the highest degradation rate, while the CSF-CS60% composite displayed the lowest degradation rate. Furthermore, a decrease in CSF content resulted in a deceleration of sponge degradation. This observation aligns with the porosity results of the sponges, which was attributed to the specific degradation characteristics of CSF by the enzyme, leading to a slower degradation rate with the mass percentage of CSF diminishing. In addition, the sponges with a high mass percentage of CS and a high porosity had reduced enzyme degradation rate [16]. Hence, by appropriately regulating the content of CS, the negative effect of the porous sponges’ rapid degradation rate was reduced, allowing the sponges’ biodegradation rate to be perfectly matched with the pace of tissue regeneration.

### 3.2. Bacterial Inhibition Rate and Drug Release

Ideal tissue-engineering sponges should possess a range of essential characteristics, including a highly hydrophilia three-dimensional structure, efficient material exchange capacity, excellent biodegradability and superior mechanical properties, as well as high efficiency in terms of biocompatibility and strength [41]. Drug-loaded release and bacterial inhibition rates are the main focuses for the practical application of composite sponges for tissue engineering. The potential of CSF-CS composite sponges in practical applications was explored by investigating in vitro drug-carrying release and bacterial inhibition rates.

#### 3.2.1. Drug Release

The absorbance of the calibration solution (with a concentration range from 0.0 to 400 μg/mL) was determined using a UV–visible spectrophotometer. The concentration (x) of IBU was utilized as the independent variable, while the absorbance (y) served as the dependent variable for plotting in order to derive the equation of the standard curve for the IBU solution: y = 0.00158645x + 0.0695208, with a correlation coefficient of r^2^ = 0.9609.

**Figure 7:**
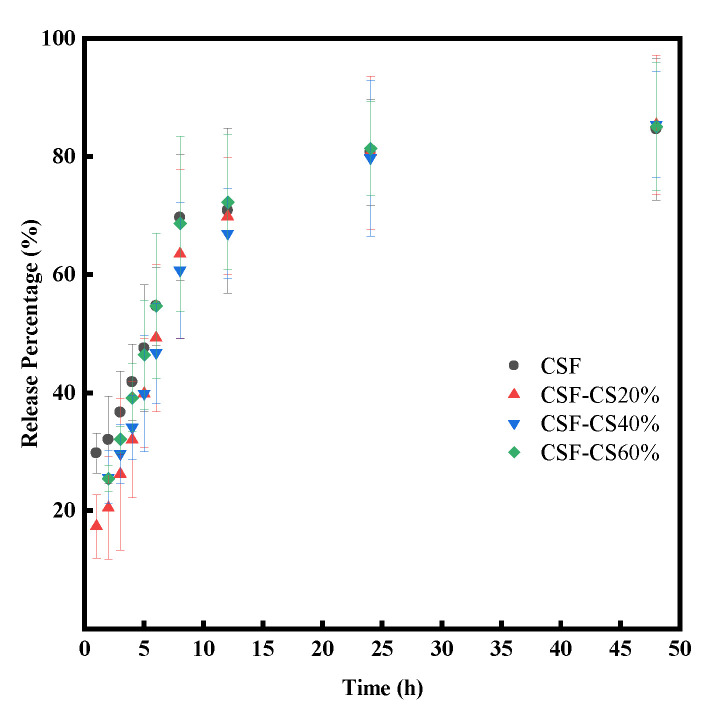
Drug release in PBS (pH = 7.4) for the different mass percentages of CSF-CS sponges at 37 °C.

Localized drug delivery curve: the release rate of the drug localized by CSF-CS compo-site sponges is shown in Figure 7. As shown in Figure 7, the drug’s release rate exhibited an initial increase followed by a plateauing trend.

IBU absorbed into the sponge through physical adsorption. The primary mechanism for the release of IBU molecules is divided into two components. In the first phase, the concentration gradient causes the IBU molecules to swiftly diffuse from the sponge to the PBS. With time, the concentration of IBU in the sponge decreases, resulting in a decreased concentration differential from the PBS solution. This limits the IBU molecule diffusion and slows its release. After about 50 h, the drug release is roughly balanced. The drug release rate impacts wound healing, ensuring stable drug concentration and sustained action at the wound site, which both avoids adverse reactions and guarantees therapeutic effects. An appropriate drug release rate facilitates wound healing. In practical applications, it is necessary to select an appropriate drug delivery system based on the type of drug and wound, and optimize the release rate accordingly.

#### 3.2.2. Antibacterial Tests: Inhibitory Bacteria Tests

**Figure 8:**
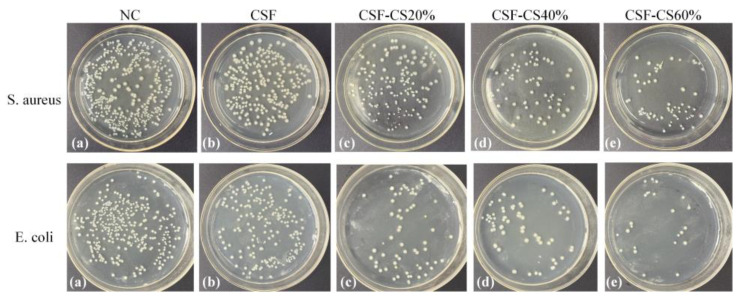
Bacterial inhibition rates for the different mass percentages of CSF-CS composite sponges. (**a**) Blank sample, (**b**) CSF, (**c**) CSF-CS20%, (**d**) CSF-CS40% and (**e**) CSF-CS60%.

**Figure 9:**
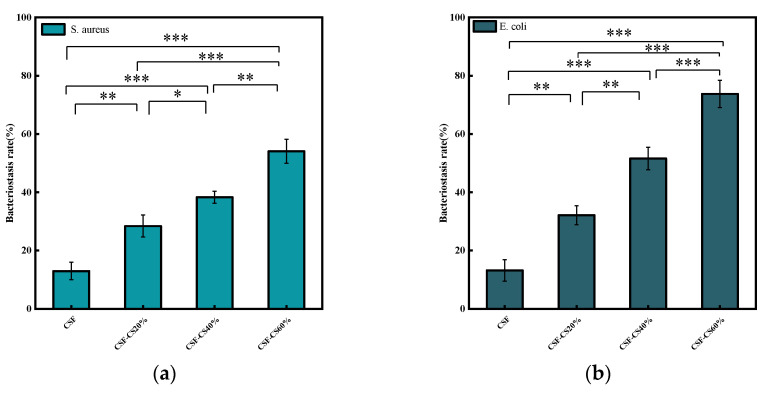
Bacterial inhibition rate for the different mass percentages of CSF-CS composite sponges. (**a**) *S. aureus*, (**b**) *E. coli*. Confidence level is labeled as: * indicates *p* < 0.05, ** indicates *p* < 0.01, and *** indicates *p* < 0.001.

In order to evaluate the bacterial inhibition performance of CSF-CS composite sponges, *E. coli* was used as a representative Gram-negative bacterium and *S. aureus* as a representative Gram-positive bacterium. The inhibition rates of the sponges against *E. coli* and *S. aureus* are shown in Figure 8 and Figure 9. The results demonstrate that the CSF-CS composite sponges in the experimental group exhibited a significant inhibitory effect on both *E. coli* and *S. aureus*. Specifically, the CSF-CS sponges effectively inhibit *E. coli* by 73.8% and *S. aureus* by 54.1%. The inhibitory effect of the sponges was enhanced as compared to the pure CSF sponge. The main reason for the difference in the inhibition rates of *E. coli* and *S. aureus* is the different mechanisms of cell wall destruction by CS on Gram-negative and Gram-positive bacteria [27]. The experiments showed that the inhibition rates of CSF-CS sponges were enhanced to different degrees by the mass percentage of CS. In the early stages of injury, bacterial infection is likely to cause inflammation, which is not conducive to wound healing. The sponges have demonstrated antibacterial properties against *E. coli*, indicating their potential for medical applications in wounds that may cause *E. coli* infections. 

## 4. Conclusions

The formation of a stable β-sheet structure through hydrogen bonding interactions was observed in CSF and CS sponge, with the morphology, porosity, and internal structure of the sponges being characterized. In order to address the poor elastic restoring force of pure CS-based sponges, it was proposed to broaden the application of the CSF silk solution in the field of functional wound dressing with localized drug delivery. In this investigation, the physicochemical and antimicrobial properties of CSF-CS sponges were prepared by the freeze-drying method, and the main conclusions are as follows: The prepared sponges exhibited a crosslinked matrix structure confirmed by changes in the XRD diffraction peaks and FTIR results. These sponges exhibited favorable mechanical properties, porosity and swelling, with an increase in the mass percentage of CS in the sponges, an increase in swelling of up to 400%, and porosity above 70%. The sponges had good absorption and swelling rates, suitable pore sizes, good biocompatibility, and superior mechanical properties, which show their great application prospects in wound dressings. This investigation showed that the drug release rate of CSF-CS composite sponges reached about 60% at 10 h. CSF-CS composite sponges showed good bacterial inhibition with *E. coli* and *S. aureus*. In conclusion, these CSF-CS sponges show great potential as wound dressings and have potential applications as a localized drug release system. Further in vivo experiments are currently underway.

## Figures and Tables

**Table 1:** Porosity and mean pore size of different mass percentages of CSF-CS composite sponges.

Sample	Mean Pore Size (μm)	Porosity (%)
CSF	126.2 ± 9.6	60.5 ± 4.2
CSF-CS20%	85.1 ± 13.7	71.2 ± 3.7
CSF-CS40%	81.9 ± 5.3	83.0 ± 2.4
CSF-CS60%	60.2 ± 5.9	84.7 ± 5.6

## Data Availability

Data are contained within the article.
